# An examination of sexual dysfunction in a cohort of women cancer survivors

**DOI:** 10.1080/28352610.2025.2540623

**Published:** 2025-09-26

**Authors:** Pearman Parker, Emily Hallgren, Reid D. Landes, Traci A. Owen, Pearl A. McElfish

**Affiliations:** a College of Nursing, University of Arkansas for Medical Sciences, Little Rock, AR, USA; b Larner College of Medicine, University of Vermont, Burlington, VT, USA; c College of Medicine, University of Arkansas for Medical Sciences, Little Rock, AR, USA; d Intimate Pathways Center for Sexual Health, Broken Arrow, OK, USA; e UAMS College of Medicine, University of Arkansas for Medical Sciences Northwest, Springdale, AR, USA

**Keywords:** Sexual dysfunction, cancer survivorship, reproductive cancers, non-reproductive cancers

## Abstract

Sexual dysfunction (i.e. sexual side effects) related to cancer and cancer treatment is a common challenge for women in survivorship. Most research has focused on women with a history of reproductive cancers, leaving sexual side effects among survivors of non-reproductive cancers understudied. Using data from a cancer survivor needs assessment, we sought to better understand the prevalence of sexual side effects among women cancer survivors. We summarized the prevalence of sexual side effects among women with a history of reproductive and non-reproductive cancers. Among the 335 women who completed the survey, about half (49.8%) had a history of reproductive cancers and the other 50.2% had a history of non-reproductive cancers. Survivors in both groups reported the presence of sexual dysfunction at the same rate of 19%, with 86% of those women attributing the effects to their cancer or treatment. However, only 15% of those with sexual dysfunction sought treatment. Women survivors who reported sexual dysfunction had a lower quality of overall health, mental health, and quality of life compared to the women who did not have sexual side effects. Our findings support the need for developing approaches to manage sexual side effects in survivorship regardless of cancer type.

Sexual dysfunction resulting from cancer and cancer therapies is a troublesome, yet common occurrence for most women cancer survivors [1]. While sexual dysfunction is most commonly known to affect women with a history of reproductive cancers, evidence suggests these side effects extend to survivors of non-reproductive cancers [2]. Side effects such as vaginal dryness, loss of arousal, loss of desire, and changes in body image can present persistent challenges in survivorship regardless of cancer type [3–6]. Additionally, sexual health side effects can affect other aspects in survivorship including mental health and quality of life [7].

Most of the research and interventions focused on sexual health and sexual dysfunction in cancer survivorship are focused only on reproductive cancers [8]. As a result, researchers may not be capturing the full scope of cancer survivors negatively impacted by sexual health issues. Furthermore, health care providers may also be less likely to assess for sexual health dysfunction or discuss sexual health among non-reproductive cancer survivors [9,10].

To better understand the prevalence of sexual dysfunction among women cancer survivors, we sought to: (1) document the prevalence of sexual side effects in both reproductive and non-reproductive cancers using survey results from women cancer survivors; and (2) examine how the presence of sexual side effects may relate to overall health, quality of life, and mental health.

## Materials & methods

### Sample

This was a descriptive cross-sectional study of cancer survivors who mostly lived in a rural state of Arkansas in the United States. Patients who received a cancer diagnosis and/or cancer treatment at the University of Arkansas for Medical Sciences hospital system within the past three years (excluding the past six months to minimmize patient burden), 18 years or older, and could read/write in English or Spanish were eligible for the study. Given the sexual dysfunction differences between women and men [11], we excluded men from the current analysis.

### Procedure

Patients were identified using electronic medical records and were recruited via postal mail, email, and/or text messaging. Participants provided informed consent and completed a cancer survivor survey from September 2022 to August 2023. Surveys were completed by mail, online (REDCap) [12,13], or over the telephone depending on the participants’ preferences. Each participant received a $40 gift card after completing the survey.

This study was approved by the Institutional Review Board (Protocol #274075) at the University of Arkansas for Medical Sciences.

### Measures

Participants completed the cancer survivor survey which consisted of one core survey with questions on a range of topics including physical health, mental health, and quality of life, and were randomized to complete one of three ending modules (A, B, or C). For the purposes of this paper, our sample was composed of participants who completed Module C, which included questions about sexual side effects. Additional information about study procedures can be found elsewhere [14].

We used validated questions commonly used in population-based surveys and health research [15,16] to assess quality of life (“In general, would you say your quality of life is”), physical health (“In general, would you say your health is”), and mental health (“In general, how would you rate your mental health, including your mood and your ability to think?”). Each of these items was measured on a 5-point Likert scale (Excellent, Very Good, Good, Fair, or Poor) where higher values indicate better physical heath or quality of life. We define “low” physical health or quality of life as those responses that took values of 1 (poor) or 2 (fair); otherwise, we considered the physical health or quality of life as “at least adequate.” We chose to dichotomize these quality measures emphasizing low quality, rather than high quality, since most treatments and therapies focus on improving poor outcomes rather than maintaining good ones.

### Analysis

Our statistical objective was to provide summary statistics; we did not perform hypothesis tests. Hence, we cross-tabulated the dichotomized factors: cancer type (reproductive, non-reproductive), sexual side effects (present, absent), and quality measures (low, at least adequate). The numbers of responding participants differed by the particular measure; data and SAS code use to produce the results are available in the Supplementary Material.

## Results

A total of 335 women cancer survivors completed the survey. Half (49.9%) of the participants reported a recent reproductive cancer diagnosis. Age at diagnosis was similar between women with reproductive cancers and women with non-reproductive cancer: 55 (IQR: 45–66) and 53 (IQR: 42–64), respectively. About 34% (*n* = 115) of women cancer survivors lived in rural areas as determined by the Rural-Urban Continuum Codes [17]. This was consistent with the geographic breakdown of Arkansas in which about 39% of residents lived in rural areas [18].

The majority of survivors (*n* = 307, 92%) answered the sexual side effects questions. Survivors of both reproductive and non-reproductive cancers reported the presence of sexual side effects at the same rate of 19% in both groups (29 of 152 reproductive cancer survivors and 29 of 155 non-reproductive cancer survivors who answered the question).

Of the 58 women who experienced sexual side effects overall, a large majority (>75%) in both reproductive and non-reproductive cancers attributed sexual side effects to their cancer or its treatment. However, the attribution by women diagnosed with reproductive cancer was about 15 percentage points higher than by women with a history of non-reproductive cancer: 93% and 78% respectively. Only 15% of those with sexual side effects reported receiving any treatment for the issue (7% of respondents with reproductive cancers (*n* = 27 respondents) and 21% with non-reproductive cancers (*n* = 28 respondents)). Notably, about 33% (*n* = 19) of those with sexual side effects lived in rural areas.

Compared to women who reported no sexual side effects (*n* = 235), women reporting sexual side effects (*n* = 58) reported low quality of overall health, mental health, and quality of life (overall health: 38% versus 20%; mental health: 28% versus 14%; quality of life: 17% versus 9%).

## Discussion

We found that about one fifth of women survivors of both reproductive and non-reproductive cancers reported sexual side effects. More than three quarters of the women who reported sexual side effects attributed the symptoms to their cancer or its treatment regardless of cancer type. These findings were consistent with other studies documenting sexual dysfunction among survivors of reproductive and non-reproductive cancers, though the dysfunction rates between cancer types varied among studies [19,20]. Specifically, Seguin and others (2020) found no major differences in sexual health deterioration (a concept similar to sexual dysfunction) among reproductive and non-reproductive cancer survivors in France five years after a diagnosis. A little more than half of the participants reported sexual health deterioration [20], which is higher than our findings of 19% of cancer survivors experiencing sexual side effects. Our findings were similar to Mutsch and colleagues (2019) who found approximately 30% of 577 adolescents and young adult cancer patients reported sexual dissatisfaction (a concept related to sexual dysfunction) with little difference between reproductive and non-reproductive cancer types [21]. We suspect the main reasons for the minimal differences among reproductive and non-reproductive cancers is related to the similarities of the sequela of cancer treatment (i.e. toxicity effects on body, reduced ability to connect, partner issues, or relational stress). In addition to aligning with the results of these prior studies, this report contributes to the knowledge regarding women cancer survivors because few cohort studies compare sexual health side effect differences between reproductive and non-reproductive cancers in women. Our study specifically examined sexual health during survivorship in a sample of adult women who mostly lived in a rural state in the United States – an often-understudied area of research.

Additionally, in our sample, proportionately more women cancer survivors with sexual side effects experienced low quality of life, mental health, and overall health than those without sexual side effects. The findings were consistent with the both qualitative and quantitative research documenting the negative effect of cancer treatment on sexual health and the resulting impacts on quality of life, mental health, and overall health [6,14,22–24]. Brajkovic and others (2021) examined a related concept – sexual quality of life – among breast cancer survivors in a systematic review. The authors found significant evidence that breast cancer treatment negatively affected body image, self-esteem, and social support which reduced sexual quality of life [25]. This report extends our knowledge of possible relationships of sexual side effects with quality of life, mental health, and overall health among women cancer survivors of non-reproductive cancers and reinforces the same in those surviving reproductive cancers.

Our findings document the prevalence of sexual side effects in a sample of survivors of multiple cancer types and the interplay of sexual side effects with quality of life, mental health, and overall health. Our findings also provide additional evidence for developing approaches to manage sexual side effects in survivorship. The American Society of Clinical Oncology (ASCO) recognizes this barrier in survivorship care and provides clinical guidelines for oncology care teams managing sexual side effects of women (and men) cancer survivors [26].

Conversations surrounding sexual health (including any sexual side effects) should be initiated by health care team members, start at diagnosis, and continue through survivorship [26]. Discussions are warranted since most (85%) of our sample of women cancer survivors who reported sexual side effects also reported not receiving treatment for sexual concerns. In particular, ASCO guidelines recommend psychosocial or psychosexual counseling as another treatment modality for such mind–body domains [26]. These counseling therapies may not be a priority for providers due to geographical or budget restrictions – a prevalent problem for providers in rural areas. Instead, nursing staff may be the best facilitators to initiate sexual health conversations, provide support, and guide patients through survivorship. Fostering communication in the clinical setting may be invaluable avenues to support women cancer survivors in their sexual health and mental health, physical health, overall quality of life in survivorship.

## Limitations

Our study was not without limitations. First, while we identified that 15% of those with sexual side effects reported receiving treatment, we did not collect data on the type of treatment. Second, we also were unable to determine reasons (if any) for the lack of sexual side effect treatment. Third, we were not able to determine the impact of rurality on accessing sexual health treatment because we did not have access to detailed data on the geographic barriers to sexual health or cancer treatment centers. Despite these limitations, this descriptive paper provides an overview of the prevalence of sexual side effects in a cohort of women cancer survivors in both reproductive and non-reproductive cancers.

## Future research

Future research should examine the desire, openness, and accessibility for sexual health therapies among women cancer survivors in both rural and urban settings. Significant differences in preferences for delivery of treatment (i.e. telehealth, in-person visits) may vary. Identifying these differences could inform the creation of tailored clinical programs to best suit the sexual health needs of various populations. Lastly, future research should examine the interplay of other variables beyond overall health, mental health, and quality of life on sexual health. Factors such as social determinants of health, socioeconomic status, and history of childhood sexual abuse could have a significant impact on cancer survivors’ sexual health.

## Supplementary Material

Supp 1

## References

[1] Office of Population Affairs. Reproductive Cancers. n.d. Available from: https://opa.hhs.gov/reproductive-health/reproductive-cancers.

[2] Wettergren L, Eriksson LE, Bergström C, Prevalence and risk factors for sexual dysfunction in young women following a cancer diagnosis – a population-based study. Acta Oncol. 2022;61(10):1165–1172. doi:10.1080/0284186X.2022.2112283 36176069

[3] Marsh S, Borges VF, Coons HL, Sexual health after a breast cancer diagnosis in young women: clinical implications for patients and providers. Breast Cancer Res Treat. 2020;184(3):655–663. doi:10.1007/s10549-020-05880-3 32968951

[4] Reese JB, Sorice K, Lepore SJ, Patient-clinician communication about sexual health in breast cancer: a mixed-methods analysis of clinic dialogue. Patient Educ Couns. 2019;102(3):436–442. doi:10.1016/j.pec.2018.10.003 30314828 PMC6421101

[5] Black KZ, Eng E, Schaal JC, The other side of through: young breast cancer survivors’ spectrum of sexual and reproductive health needs. Qual Health Res. 2020;30(13):2019–2032. doi:10.1177/1049732320929649 32552407 PMC10557425

[6] Valpey R, Kucherer S, Nguyen J. Sexual dysfunction in female cancer survivors: a narrative review. Gen Hosp Psychiatry. 2019;60:141–147. doi:10.1016/j.genhosppsych.2019.04.003 31030966

[7] Schover LR. Sexual quality of life in men and women after cancer. Climacteric. 2019;22(6):553–557. doi:10.1080/13697137.2018.1526893 30380961

[8] Sousa Rodrigues Guedes T, Barbosa Otoni Gonçalves Guedes M, de Castro Santana R, Sexual dysfunction in women with cancer: a systematic review of longitudinal studies. Int J Environ Res Public Health. 2022;19(19):1–15. doi:10.3390/ijerph191911921 PMC9564951 36231221

[9] Canzona MR, Garcia D, Fisher CL, Communication about sexual health with breast cancer survivors: variation among patient and provider perspectives. Patient Educ Couns. 2016;99(11):1814–1820. doi:10.1016/j.pec.2016.06.019 27387120

[10] Canzona MR, Fisher CL, Wright KB, Talking about sexual health during survivorship: understanding what shapes breast cancer survivors’ willingness to communicate with providers. J Cancer Surviv. 2019;13(6):932–942. doi:10.1007/s11764-019-00809-2 31741248

[11] Lewis RW, Fugl-Meyer KS, Corona G, Definitions/epidemiology/risk factors for sexual dysfunction. J Sex Med. 2010;7(4 Pt 2):1598–1607. doi:10.1111/j.1743-6109.2010.01778.x 20388160

[12] Harris PA, Taylor R, Minor BL, The REDCap consortium: building an international community of software platform partners. J Biomed Inform. 2019;95:103208. doi:10.1016/j.jbi.2019.103208 31078660 PMC7254481

[13] Harris PA, Taylor R, Thielke R, Research electronic data capture (REDCap) – a metadata-driven methodology and workflow process for providing translational research informatics support. J Biomed Inform. 2009;42(2):377–381. doi:10.1016/j.jbi.2008.08.010 18929686 PMC2700030

[14] Moore R, Hallgren E, Worrell S, I’m learning to live after cancer and its treatment”: exploring the challenges of cancer survivorship in Arkansas. SSM – Qualitative Research in Health. 2025;7:100519. doi:10.1016/j.ssmqr.2024.100519 40575724 PMC12199791

[15] HealthMeasures. List of Adult Measures. 2025. Available from: https://www.healthmeasures.net/explore-measurement-systems/promis/intro-to-promis/list-of-adult-measures.

[16] Centers for Disease Control and Prevention. Behavioral Risk Factor Surveillance System. 2024. Available from: https://www.cdc.gov/brfss/index.html.

[17] U.S. Department of Agriculture. Rural-Urban Continuum Codes – Documentation. (2025). Available from: https://www.ers.usda.gov/data-products/rural-urban-continuum-codes/documentation.

[18] Rural Health Information Hub. Arkansas. 2025. Available from: https://www.ruralhealthinfo.org/states/arkansas.

[19] Ussher JM, Perz J, Gilbert E. Perceived causes and consequences of sexual changes after cancer for women and men: a mixed method study. BMC Cancer. 2015;15:268. doi:10.1186/s12885-015-1243-8 25885443 PMC4407322

[20] Seguin L, Touzani R, Bouhnik AD, Deterioration of sexual health in cancer survivors five years after diagnosis: data from the French national prospective VICAN survey. Cancers (Basel). 2020;12(11):1–21. doi:10.3390/cancers12113453 PMC7699784 33233583

[21] Mütsch J, Friedrich M, Leuteritz K, Sexuality and cancer in adolescents and young adults – a comparison between reproductive cancer patients and patients with non-reproductive cancer. BMC Cancer. 2019;19(1):828. doi:10.1186/s12885-019-6009-2 31438895 PMC6704507

[22] Webber K, Mok K, Bennett B, If I am in the mood, I enjoy it: an exploration of cancer-related fatigue and sexual functioning in women with breast cancer. Oncologist. 2011;16(9):1333–1344. doi:10.1634/theoncologist.2011-0100 21835897 PMC3228173

[23] Wettergren L, Ljungman L, Micaux Obol C, Sexual dysfunction and fertility-related distress in young adults with cancer over 5 years following diagnosis: study protocol of the Fex-Can cohort study. BMC Cancer. 2020;20(1):722. doi:10.1186/s12885-020-07175-8 32758179 PMC7409491

[24] Yip KH, Yip YC, Tsui WK, Navigating changes: a qualitative study exploring the health-related quality of life of breast cancer survivors during the coronavirus disease 2019 pandemic. J Adv Nurs. 2024;80(4):1531–1544. doi:10.1111/jan.15909 37902114

[25] Brajkovic L, Sladic P, Kopilaš V. Sexual quality of life in women with breast cancer. Health Psychol Res. 2021;9(1):24512. doi:10.52965/001c.24512 34746481 PMC8567769

[26] Carter J, Lacchetti C, Andersen BL, Interventions to address sexual problems in people with cancer: American society of clinical oncology clinical practice guideline adaptation of cancer care Ontario guideline. J Clin Oncol. 2018;36(5):492–511. doi:10.1200/JCO.2017.75.8995 29227723

