# Supplementary material for: An examination of sexual dysfunction in a cohort of women cancer survivors
Source: Cancer Surviv Res Care. Author manuscript; Available in PMC 2026 May 1. (PMC13132494; doi:10.1080/28352610.2025.2540623)
Supplement: Supp 1 [file NIHMS2120191-supplement-Supp_1.docx]

Supplementary Material

for

An examination of sexual dysfunction in a cohort of women cancer survivors

by

Parker P, Hallgren E, Landes RD, Owen TA, & McElfish PA

/*--------------------------------------------------------------------------------

--------------------------------------------------------------------------------

--------------------------------------------------------------------------------

This SAS code analyzes the data used for the paper and produces results in

the order of results presented in the paper.

The code is organized in "chunks" with short descriptors before each.

This was prepared by Reid D. Landes (rdlandes@uams.edu) in May 2025.

--------------------------------------------------------------------------------

--------------------------------------------------------------------------------

--------------------------------------------------------------------------------*/

/*================================================================================

000 - Options that make the Output and Log windows easier to view.

================================================================================*/

options ls = **100** ps = **55** nodate nonumber nonotes formdlim = " ";

/*================================================================================

010 - Importing the data

================================================================================*/

/* Set the LOCATION of where the datafile,

"Sexual_dysfunction_in_cancer_survivors_Working_data.xlsx", is stored.

Replace the "~~D:\MyFolder\MySubfolder~~" with your location.*/

%let LOCATION = ~~D:\MyFolder\MySubfolder~~ ;

/* Importing the data. */

**proc** **import**

out = WORKING_DATA

datafile = "&LOCATION\Sexual_dysfunction_in_cancer_survivors_Working_data.xlsx"

dbms = xlsx

replace;

sheet = "Data";

**run**;

**quit**;

/*================================================================================

020 - Proportions of survivors in each cancer type

And age-at-diagnosis summaries

================================================================================*/

title1 "Cancer survivors by cancer type";

title2 "Non-reproductive (0) & Reproductive (1)";

**proc** **freq** data = WORKING_DATA;

tables REPRO_CAN;

**run**;

title1 "Age at diagnosis by cancer type";

title2 "Non-reproductive (0) & Reproductive (1)";

**proc** **means** data = WORKING_DATA mean median q1 q3 maxdec=**0**;

class REPRO_CAN;

var AGE_DX;

**run**;

/*================================================================================

030 - Cross tablulations of sexual side effects with cancer type

================================================================================*/

/* Values of SSE0:

1 - No to side effects

2 - Yes to side effects

3 - Doesn't apply

8 - Don't know / unsure */

title1 "Prevalence of those with sexual side effects";

title2 "By cancer type";

**proc** **freq** data = WORKING_DATA;

tables REPRO_CAN*SSE0 / nocol nopercent;

**run**;

/* This analysis is limited to only those who said 'Yes' to side effects.

Values of SSE1:

1 - No to related to cancer or its treatment

2 - Yes to related to cancer or its treatment

8 - Don't know / Not sure

9 - Prefer not to answer */

title1 "Sexual side effects caused by cancer (identified by SSE1 = 2)";

title2 "By canncer type";

**proc** **freq** data = WORKING_DATA;

where SSE0 = **2**;

tables REPRO_CAN*SSE1 / nocol nopercent;

**run**;

title1 "Sought treatment for Sexual side effects";

title2 "By cancer type";

**proc** **freq** data = WORKING_DATA;

where SSE0 = **2**;

tables REPRO_CAN*SSE2/ nocol nopercent;

**run**;

/*================================================================================

035 - Cross tablulations of sexual side effects with rurality

================================================================================*/

title1 "Rurality of Sample";

**proc** **freq** data = WORKING_DATA;

tables RURAL ;

**run**;

/* Values of SSE0:

1 - No to side effects

2 - Yes to side effects

3 - Doesn't apply

8 - Don't know / unsure */

title1 "Prevalence of those with sexual side effects (SSE0 = 2)";

title2 "By rurality";

**proc** **freq** data = WORKING_DATA;

tables RURAL*SSE0 / norow nopercent;

**run**;

/*================================================================================

040 - Cross tablulations of quality of life measures

with presence/absence of SSEs

================================================================================*/

/* This analysis is focused on those who reported no sexual side effects (SSE0 = 1)

and those who did (SSE0 = 2). We crosstabulate by the quality of health, mental

health, and life variables. On these quality variables, we focus on those who

report lower quality ("neg") and at least adequate quality ("pos"). */

title1 "Sexual side effects";

title2 "associated with QOL measures";

**proc** **freq** data = WORKING_DATA;

tables SSE0*( HEALTH_pn MENTAL_HLTH_pn LIFE_pn) / nocol nopercent;

**run**;
